# Luteolin Synergistically Enhances Antitumor Activity of Oxaliplatin in Colorectal Carcinoma via AMPK Inhibition

**DOI:** 10.3390/antiox11040626

**Published:** 2022-03-24

**Authors:** Chan Ho Jang, Nayoung Moon, Jinkyung Lee, Min Jeong Kwon, Jisun Oh, Jong-Sang Kim

**Affiliations:** 1 School of Food Science and Biotechnology, Kyungpook National University, Daegu 41566, Korea; cksghwkd7@gmail.com (C.H.J.); moonna1008@naver.com (N.M.); wlsrud9526@naver.com (J.L.); rnjsalswjd1005@naver.com (M.J.K.); 2 New Drug Development Center, Daegu-Gyeongbuk Medical Innovation Foundation, Daegu 41061, Korea

**Keywords:** luteolin, oxaliplatin, colorectal cancer, xenograft tumor, AMPK inhibition

## Abstract

Luteolin is a naturally-occurring polyphenolic compound that is known to have antioxidative and antitumor activities in vitro. This study aimed to examine the in vivo anticancer efficacy of luteolin in conjunction with oxaliplatin treatment using a colorectal carcinoma xenograft mouse model. HCT116 human colorectal carcinoma cells were subcutaneously implanted into BALB/c nude mice, followed by the intraperitoneal administration of luteolin at a dose of 50 mg/kg body weight (BW)/day with or without oxaliplatin at a dose of 10 mg/kg BW/day three times per week for a total of 3 weeks. The combined luteolin and oxaliplatin treatment resulted in the synergistic suppression of the growth of HCT116 xenograft tumors when compared to treatment with luteolin or oxaliplatin alone. In addition, the combined treatment significantly increased the expression of cleaved PARP and p53 in the xenograft tumors compared with the vehicle control, but only marginally affected the level of heme oxygenase-1 (HO-1). These results indicated that luteolin treatment retarded oxaliplatin-induced tumor growth by facilitating apoptotic cell death and inhibiting HO-1-mediated cytoprotection. Therefore, these findings suggest the synergistic potential of dietary luteolin in conjunction with conventional chemotherapy for the treatment of colorectal cancer.

## 1. Introduction

Colorectal cancer is the third most frequently diagnosed cancer and the second leading cause of cancer mortality globally [1]. There is now a large body of epidemiologic evidence showing that the increased incidence of colorectal cancer is associated with low intake of fruits and vegetables, high intake of red and processed meat, and a lack of physical activity [2,3,4,5].

Several flavonoids that are abundant in fruits and vegetables are reportedly capable of reducing oxidative stress and stimulating cellular defense mechanisms through the activation of the nuclear factor erythroid-derived 2-like 2 (Nrf2)/antioxidant response element (ARE) signaling pathway, thereby preventing the initiation of various cancers [6,7,8]. Recent studies have shown that several dietary flavonoids with antioxidant activity can inhibit tumor occurrence or development [9,10]. Based on these reports, many cancer patients receiving chemotherapy have been surveyed to consume antioxidant phytochemicals in the hope of inducing a synergistic effect with the conventional chemotherapy [11,12]. However, many studies have reported that cancer cells can take advantage of the activation of the Nrf2/ARE signaling pathway to resist chemotherapy by attenuating the pro-oxidant and cytotoxic activities of chemotherapeutic agents [13,14]. For example, the strong induction of heme oxygenase-1 (HO-1), one of the Nrf2/ARE downstream antioxidant/detoxifying enzymes, favors tumorigenesis and cancer progression [15,16]. Therefore, the intake of phytochemicals that can stimulate the Nrf2/ARE/HO-1 axis should be carefully considered due to its role as a “double-edged sword” [13].

Luteolin is a bioactive flavonoid abundant in a variety of fruits and vegetables, including radicchio, broccoli, pepper, thyme, and chicory. It is well established that this flavonoid can activate the Nrf2 signaling pathway and prevent azoxymethane-induced colorectal carcinogenesis in mice [17,18]. In addition, our previous in vitro study revealed that luteolin potentiated the anticancer activity of oxaliplatin, one of the most commonly used chemotherapeutic drugs for colorectal cancer, by modulating the oxaliplatin-induced cell cycle arrest of HCT116 human colorectal carcinoma cells into apoptotic cell death [19]. Despite its potential to induce HO-1, luteolin decreased p21 protein expression in p53-expressing HCT116 cells, decreased oxaliplatin-induced cell cycle arrest, and increased apoptotic cell death [19]. Thus, it was expected that luteolin would act synergistically in oxaliplatin-treated individuals to suppress colorectal tumor growth.

In the present study, we examined the anticancer effectiveness of luteolin in oxaliplatin-treated mice xenografted with HCT116 cells. The findings of this study may provide insights into the impact of the dietary intake of luteolin or luteolin-containing foods during conventional chemotherapy for colorectal cancer.

## 2. Materials and Methods

### 2.1. Chemicals

Commercially available luteolin (Purity, >98% by HPLC) and oxaliplatin (Purity, >99% by HPLC) were purchased from Biopurify Phytochemicals, Ltd. (Chengdu, China) and LC Laboratories (Woburn, MA, USA), respectively. Both chemicals were dissolved in dimethyl sulfoxide (DMSO; Sigma-Aldrich, Inc., St. Louis, MO, USA), and stock solutions of 25 mmol/L were prepared.

### 2.2. Cell Lines and Culture

The HCT116 human colorectal carcinoma cell line was obtained from the Korean Cell Line Bank (Seoul, Korea). HCT116-ARE cells carrying the ARE-luciferase reporter gene were generated as previously described [19]. Both cell lines were maintained in Dulbecco’s modified eagle medium (Welgene, Inc., Gyeongsan, Korea) supplemented with 10% (*v*/*v*) fetal bovine serum (FBS; Welgene, Inc.), 1% (*v*/*v*) penicillin-streptomycin (HyClone Laboratories, Inc., a part of Cytiva, Marlborough, MA, USA), 2% (*v*/*v*) 4-(2-hydroxyethyl)-1-piperazineethanesulfonic acid (HEPES; HyClone Laboratories, Inc.), and 1% (*v*/*v*) minimum essential medium non-essential amino acids (MEM NEAA; Gibco Thermo Fisher Scientific, Inc., Waltham, MA, USA) in a CO_2_ incubator (MCO-19 AIC, Sanyo, Osaka, Japan) at 37 °C with a humidified atmosphere containing 5% CO_2_. Upon reaching approximately 80% confluency, the cells were routinely subcultured at a seeding density of 1 × 10^5^ cells in a 100-mm culture dish (SPL Life Sciences Co., Ltd., Pocheon, Korea) using phosphate-buffered saline (PBS) and trypsin-ethylenediaminetetraacetic acid (EDTA) solution (Welgene, Inc.).

### 2.3. Cell Proliferation Assay

HCT116 cells were plated at a density of 5 × 10^3^ cells per well in a 96-well plate. After incubation for 24 h in maintenance medium, the cells were treated with 12.5 μM of luteolin in the absence or presence of 1 μM of oxaliplatin in 0.5% FBS-containing culture medium for 24, 48, and 72 h. At each time-point, cell proliferation was determined using the Cell Counting Kit-8 (CCK-8; Dojindo Laboratories, Kumamoto, Japan), as previously described [19].

### 2.4. Apoptosis Assay

HCT116 cells were plated at a density of 1 × 10^6^ cells in a 90-mm dish. After incubation for 24 h, the cells were treated with 25 μM of luteolin in the absence or presence of 1 μM of oxaliplatin in 0.5% FBS-containing culture medium for 24 h and then analyzed for apoptosis using the Fluorescein Isothiocyanate (FITC)-Annexin V Apoptosis Detection Kit I (BD Pharmingen, Inc., Franklin Lakes, NJ, USA) [19]. Briefly, the cells were harvested, washed in PBS, and resuspended in Annexin V binding buffer at a density of 1 × 10^5^ cells per 100 μL. The cell suspension was allowed to react with 5 μL of FITC-conjugated Annexin V and 5 μL of propidium iodide at 22 °C for 15 min. The stained cells were analyzed using a FACS Aria III sorter (BD Biosciences, Franklin, NJ, USA). Staurosporine (2 μM, 4 h), an apoptosis-inducing agent, was used as a positive control.

### 2.5. Animal Study

The animal study was conducted in accordance with the guidelines of the Institutional Animal Care and Use Committee of Kyungpook National University (Approval Number 2019-0060). Six-week-old male BALB/c nude mice were purchased from Orient Bio, Inc. (Seongnam, Korea) and kept under standard conditions (temperature, 22 °C ± 2 °C; relative humidity, 45% ± 5%; 12-h light-dark cycle). The mice were allowed free access to drinking water and standard mouse chow pellets (Daehan Bio Link, Eumseong, Korea).

The xenograft model was established as previously described [20]. Briefly, HCT116 cells were subcutaneously injected to both the left and right flank of each mouse (3 × 10^6^ cells per injection). When palpable tumors reached a volume of approximately 80 mm^3^ at the injection sites, mice were randomly assigned into four different treatment groups (seven tumors per group): (1) vehicle control, (2) luteolin only at a dose of 50 mg/kg body weight (BW), (3) oxaliplatin only at a dose of 10 mg/kg BW, and (4) a combination of luteolin (at 50 mg/kg BW) and oxaliplatin (at 10 mg/kg BW). Luteolin and/or oxaliplatin were dissolved in vehicle consisting of 10% (*v*/*v*) DMSO and 5% (*v*/*v*) Tween^®^ 80 (Sigma-Aldrich, Inc.) in sterile normal saline solution, and intraperitoneally injected three times per week for a total of 3 weeks (Supplementary Figure S1). The body weight of the mice and tumor size were monitored three times per week until the mice were sacrificed. The length and width of each xenograft tumor were measured using a caliper (Mitutoyo, Kawasaki, Japan). The tumor volume was calculated from the following formula: Tumor volume (mm^3^) = [Length (mm) × Width (mm)^2^]/2. After sacrifice, the xenograft tumor tissues were dissected, weighed, snap-frozen, and stored at −80 °C until use.

### 2.6. Western Blotting Analysis

The dissected tumor tissues were homogenized in pre-cooled lysis buffer (20 mM of Tris-HCl, 145 mM of NaCl, 10% glycerol, 5 mM of EDTA, 1% Triton X, and 0.5% Nonidet P-40) using a tissue homogenizer (Omni International, Kennesaw, GA, USA). The protein concentration in the lysate was quantified by the Bradford assay. Then, equal amounts of protein were separated by sodium dodecyl sulfate polyacrylamide gel electrophoresis, transferred to a polyvinylidene fluoride membrane (Merck Millipore, Burlington, MA, USA), and analyzed using specific antibodies. The primary antibodies used in this study were immunoglobulin G (IgG) against mouse p53 (#SC-126, Santa Cruz Biotechnology, Inc., Santa Cruz, CA, USA), mouse β-actin (#SC-47778, Santa Cruz Biotechnology, Inc.), rabbit HO-1 (#ab13243, Abcam, Cambridge, UK), and rabbit poly-adenosine diphosphate-ribose polymerase (PARP; #9542S, Cell Signaling Technology, Beverly, MA, USA). The secondary antibodies were anti-rabbit IgG (#31460, Thermo Fisher Scientific, Inc., Waltham, MA, USA) and anti-mouse IgG (#31430, Thermo Fisher Scientific, Inc.) conjugated to horseradish peroxidase. The primary and secondary antibodies were diluted in Tris-buffered saline containing 0.05% *v*/*v* Tween^®^ 20 (all from Sigma-Aldrich, Inc.) at a dilution ratio of 1:1000 and 1:2000, respectively. The antibody-bound proteins were visualized using the SuperSignal^®^ West Pico PLUS Chemiluminescent Substrate or SuperSignal™ West Femto Maximum Sensitivity Substrate kits (Thermo Fisher Scientific, Inc.) and digitized using an ImageQuant LAS 4000 Mini Biomolecular Imager (GE Healthcare Life Sciences, Pittsburgh, PA, USA). The protein band intensities were determined using the Image Studio Lite software version 5.2 (LI-COR Biotechnology, Lincoln, NE, USA).

### 2.7. Immunohistochemical Analysis

The xenograft tumor tissues were dissected, fixed in 10% (*v*/*v*) formalin solution, embedded into paraffin, and sectioned into 7-μm-thick slices using a microtome (RM-2125 RT; Leica, Nussloch, Germany), as previously described [21]. The sections were placed on Superfrost Plus microscope slides (Marienfeld, Lauda-Königshofen, Germany), dried at 37 °C for 12 h, and subjected to immunostaining.

For the immunochemical analysis, the tissue sections prepared on microscope slides were first deparaffinized by sequential immersion in xylene, 100% ethanol, 95% ethanol, 70% ethanol, and water. The sections were then treated with 0.5% (*v*/*v*) Triton X-100 in PBS for 5 min to retrieve the epitope. The tissue sections were rinsed with PBS and subsequently incubated in a blocking solution composed of 0.5% (*w*/*v*) bovine serum albumin and 0.2% (*w*/*v*) Triton X-100 in PBS for 1 h. The sections were then allowed to react with the primary antibody against proliferation cell nuclear antigen (PCNA)-conjugated to Alexa Fluor^®^ 488 (#SC-56 AF488, Santa Cruz Biotechnology, Inc.) that was diluted in the blocking solution to a final concentration of 2 μg/mL at room temperature for 2 h in the dark. The nuclei were counterstained with 1 μg/mL of 4′,6-diamidino-2-phenylindole dihydrocholoride (DAPI). The stained sections were mounted using mounting medium (Dako Fluorescent Mounting Medium; Dako, Glostrup, Denmark) and observed under a fluorescence microscope (Eclipse 80i, Nikon, Tokyo, Japan).

### 2.8. Terminal Deoxynucleotidyl Transferase Deoxyuridine Triphosphate (dUTP) Nick End Labeling (TUNEL) Assay

The deparaffinized sections were stained using an Apotag^®^ red in situ apoptosis detection kit (Merck Millipore) in accordance with the manufacturer’s instructions. Briefly, the sections were equilibrated in digoxigenin nucleotide-containing reaction buffer and subjected to react with the terminal deoxynucleotidyl transferase enzyme. The digoxigenin-labeled DNA fragments were visualized using rhodamine-conjugated anti-digoxigenin and observed under a fluorescence microscope.

### 2.9. In Vitro Protein Kinase Activity Measurement

The effect of luteolin on protein kinases was determined using the Kinase Selectivity Profiling System (Promega Corp., Madison, WI, USA) as per the manufacturer’s instructions. Briefly, a commercial panel of kinases, including, protein kinase Bα (AKT), AMP-activated protein kinase (combination of A1/B1/G2 subunits; AMPK), and p38α mitogen-activated protein kinase (MAPK), was allowed to react with a mixture of the specific substrate and co-factor in a given ATP-containing reaction buffer in the absence or presence of luteolin in a 384-well plate (Corning, Inc., Corning, NY, USA), and then incubated at 22 °C for 60 min. After the addition of ADP-Glo reagent and kinase detection reagent to all reactions and a subsequent incubation for 30 min, the kinase activity resulting from each reaction was determined using the GloMax Explorer System (Promega Corp.).

### 2.10. ARE-Luciferase Reporter Assay

HCT116-ARE cells were seeded at a density of 5 × 10^5^ cells per well in a 6-well plate and treated with the indicated concentrations of luteolin in the absence or presence of kinase inhibitors, including LY294002 (an inhibitor of phosphatidylinositol-3-kinase (PI3K)/AKT, 2.5 μM), compound C (an inhibitor of AMPK, 1 μM) (all purchased from Tokyo Chemical Industry Co., Ltd., Tokyo, Japan). After 24 h, the cells were harvested in an ice-cold PBS solution and analyzed using the Luciferase Assay System (Promega Corp.), as previously described [19].

### 2.11. Extracellular Flux Assay

The glycolytic capacity and mitochondrial respiration in HCT116 cells were assessed by measuring the extracellular acidification rate (ECAR) and oxygen consumption rate (OCR), respectively, using an Agilent Seahorse Extracellular Flux Analyzer (Agilent Technologies, Inc., Santa Clara, CA, USA). HCT116 cells were plated at a density of 1 × 10^4^ cells per well in an Agilent Seahorse XFp cell culture miniplate. After incubation for 24 h, the cells were treated with or without 25 μM of luteolin in 0.5% FBS-containing culture medium for 24 h. The XFp Sensor cartridge with the utility plate was hydrated overnight at 37 °C in a non-CO_2_ incubator. The XFp Sensor cartridge was then submerged in 200 μL of the XF Calibrant solution for 1 h prior to running the assay. For the ECAR measurement, the culture medium was replaced with XF Base Media supplemented with 2 mM of l-glutamine. The designated ports on the XFp Sensor cartridge were subsequently loaded with 10 mM of glucose, 1 μM of oligomycin (ATP synthase Complex V inhibitor), and 50 mM of 2-deoxy-glucose (2-DG). For the OCR measurement, the culture medium was replaced with XF Base Media supplemented with 25 mM of d-glucose, 4 mM of l-glutamine, and 1 mM of sodium pyruvate. The designated ports on the XFp Sensor cartridge were subsequently loaded with 2 μM of oligomycin, 0.5 μM of carbonyl cyanide-4 (trifluoromethoxy) phenylhydrazone (FCCP), and 0.5 μM of rotenone/antimycin A. The ECAR and OCR values were normalized to the total protein content in each well of the miniplate. The obtained data were analyzed using the Agilent Seahorse Wave Desktop Software (Agilent Technologies, Inc.).

### 2.12. Statistical Analysis

Statistical analysis was performed using IBM SPSS statistics for Windows, Version 25.0 (IBM Corp., Armonk, NY, USA). Statistical significance was assessed by one-way analysis of variance followed by Tukey′s post hoc honest significant difference test for multiple comparisons on average values or by Student’s *t*-test for a single comparison between the two averaged values. The significance level was set at *p* < 0.05 and indicated with different alphabetical letters, an asterisk, or a hashtag.

## 3. Results and Discussion

### 3.1. Luteolin Potentiated the Oxaliplatin-Induced Inhibtion of HCT116 Cell Proliferation

**Figure 1:**
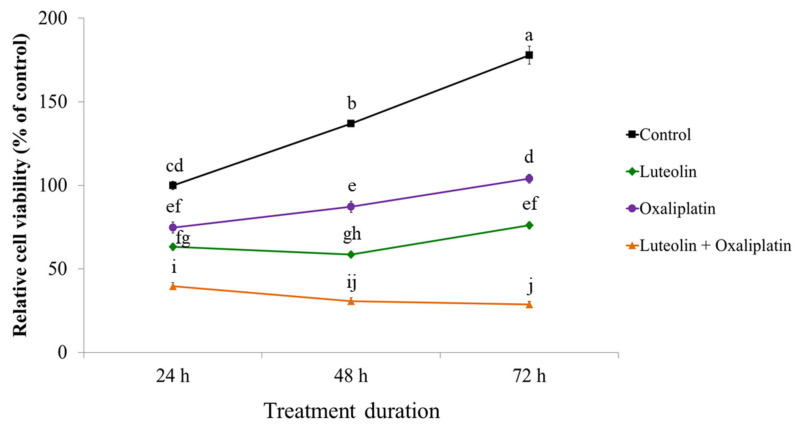
Effect of luteolin and oxaliplatin on the viability of HCT116 cells. Cells were treated with 12.5 μM luteolin in the absence or presence of 1 μM of oxaliplatin for up to 72 h. Cell viability was quantified using the CCK-8 assay. The data are expressed as the mean ± standard error of the mean (SEM) from three independent experimental sets (*n* = 3). Values marked with different letters are statistically significantly different from each other at *p* < 0.05.

The antiproliferative effects of luteolin, oxaliplatin, and their combined treatment were examined in HCT116 human colorectal carcinoma cells. The cells were treated with 12.5 μM of luteolin and 1 μM of oxaliplatin individually or in combination for up to 72 h. The viability of the treated cells was then determined at 24, 48, and 72 h (Figure 1). Compared to the control, HCT116 cell proliferation was inhibited in a time-dependent manner by luteolin or oxaliplatin alone and further suppressed by the combined treatment; cell viability after the exposure to the combined treatment for 24, 48, and 72 h was 39.7, 30.6, and 28.7% compared to the control, respectively. Moreover, the combined treatment significantly increased the percentage of apoptotic cell counts compared to the treatment with luteolin or oxaliplatin alone (Supplementary Figure S2). These observations implied that luteolin and oxaliplatin acted synergistically to cause significant suppression of HCT116 cell proliferation, which was consistent with our previous results demonstrating that luteolin facilitated the apoptotic death of HCT116 cells by releasing the cells from oxaliplatin-induced cell cycle arrest [19].

### 3.2. The Combination of Luteolin and Oxaliplatin Synergistically Suppressed HCT116 Xenograft Tumor Growth in Mice

**Figure 2:**
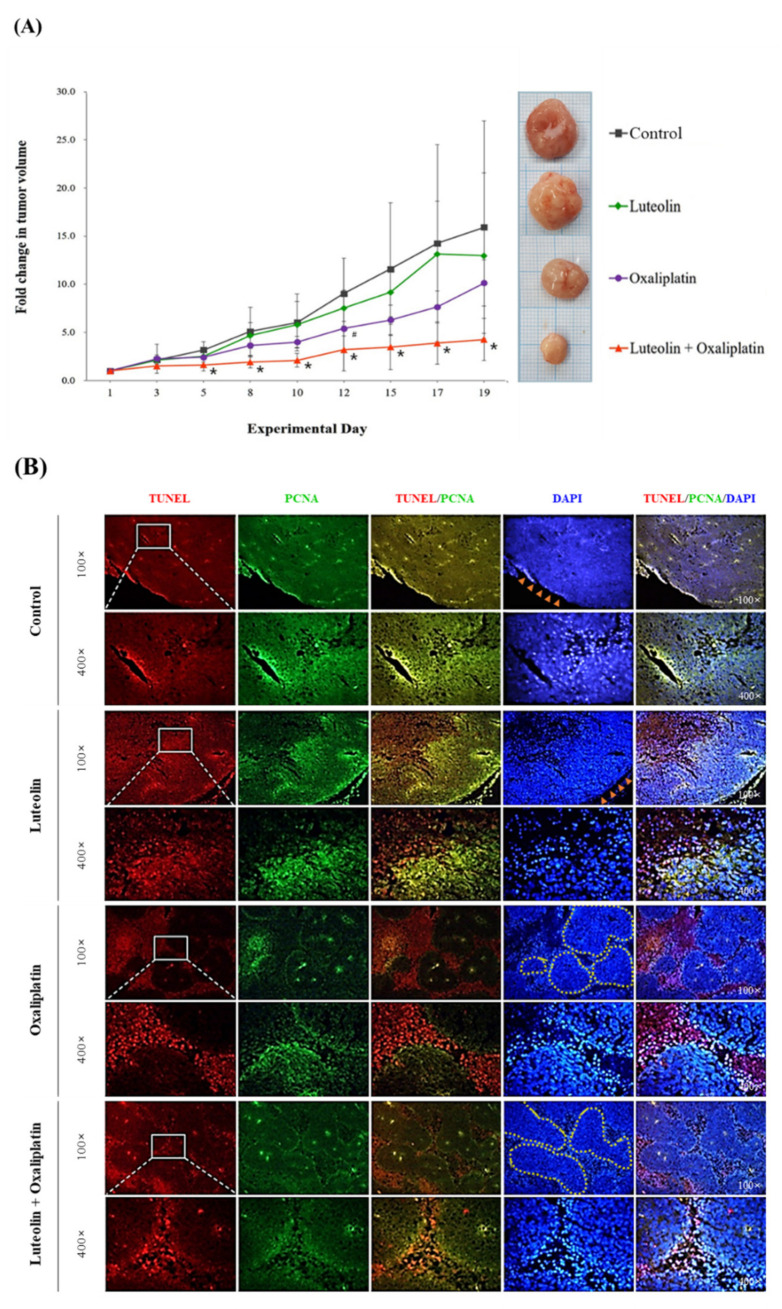
The change in volume and the immunostaining of sections of HCT116 xenograft tumors in mice treated with luteolin and oxaliplatin individually or their combination. (**A**) Fold changes in xenograft tumor volume. HCT116 tumors were xenografted in BALB/c nude mice. The mice received luteolin at 50 mg/kg BW/day and/or oxaliplatin at 10 mg/kg BW/day three times per week for 3 weeks. The tumor size was regularly measured using a caliper until the mice were sacrificed. The data are expressed as the mean ± standard deviation (SD) (*n* = 6–7 per group). A significant difference relative to the control group at *p* < 0.05 is indicated by an asterisk (*) or a hashtag (#). (**B**) Representative immunohistochemical images of the tumor sections. After the mice were sacrificed, the xenograft tumor tissue sections were dissected, sectioned, and immunostained for TUNEL (apoptosis marker; rhodamine-conjugated molecule was used for visualization, red fluorescence), PCNA (proliferation marker; FITC-conjugated IgG was used for immunostaining, green fluorescence), and DAPI (nuclear counterstain; blue fluorescence). Magnifications: 100× or 400×. Arrow heads colored in orange indicate the outer rim of tumor. Dotted lines colored in yellow indicate the cell clusters formed in the middle of the tumor mass.

The synergistic effect of luteolin and oxaliplatin against colorectal cancer was further examined using HCT116 xenograft tumors in nude mice. After the subcutaneous transplantation of HCT116 cells, tumor-bearing mice were intraperitoneally administered with luteolin alone at a dose of 50 mg/kg BW/day, oxaliplatin alone at a dose of 10 mg/kg BW/day, or their combination. The treatment was performed three times per week for a total of 3 weeks. Tumor growth was monitored, and tumor size was measured during the entire experimental period. The average tumor volume was found to gradually increase in all treatment groups over time (Figure 2A), with no significant difference in BW changes in any of the groups (Supplementary Figure S3). At the end of the experiment, the average fold increase of tumor volume in HCT116 xenograft mice treated with vehicle (control), luteolin, oxaliplatin, or the combination treatment was 15.93, 12.97, 10.13, and 4.26, respectively, compared to the first measurement of tumor volume in each group (Figure 2A). This observation demonstrated that, compared to the control, the treatment with either luteolin or oxaliplatin likely suppressed the growth of HCT116 xenograft tumors and, more importantly, that the combined luteolin and oxaliplatin treatment significantly inhibited the tumor growth. Considering that the predicted increase in tumor volume was computed to be 8.28-fold, based on a previously described formula [22], the 4.26-fold increase in tumor volume following the combined treatment indicated the synergistic inhibition of tumor growth. These findings thus indicated that luteolin augmented the anticancer activity of oxaliplatin in vivo, which was consistent with the aforementioned in vitro synergistic antiproliferative effect of luteolin and oxaliplatin.

### 3.3. Luteolin-Induced Apoptotic Cell Death in HCT116 Xenograft Tumors

To understand the possible tumor suppression mechanism of the combination treatment, immunohistochemical staining and western blotting analyses were performed. HCT116 xenograft tumor tissue sections were stained and visualized for TUNEL-positive apoptotic cells and PCNA-positive proliferating cells (Figure 2B; Supplementary Figure S4). The TUNEL-positive cells were spread throughout the tumor tissue and the PCNA-positive cells were likely located on the outer part of the tumor tissues obtained from mice treated with luteolin alone, indicating that luteolin treatment increased both the proportions of apoptotic and proliferating cells in the tumor compared to the control (Figure 2B). In contrast, the tissues from mice treated with oxaliplatin alone had multiple clusters of cells in the middle of the tumor mass and a number of TUNEL-positive cells were found mostly in interstitial-like regions in between the clusters, with some PCNA-positive cells inside the clusters (Figure 2B). These findings indicated that oxaliplatin treatment increased apoptotic cell death and presumably suppressed the outgrowth of the tumor. Interestingly, the tumors from mice treated with the combination of luteolin and oxaliplatin consisted of TUNEL-positive cells in both the interstitial-like regions and inside the cluster, with a much lower proportion of PCNA-positive cells than those found in tumor sections from other treatment groups (Figure 2B). From the observations, it can be suggested that the combined luteolin and oxaliplatin treatment potentially caused the inhibition of tumor outgrowth by increasing the proportion of cells undergoing apoptosis rather than proliferation at the periphery of the tumor mass as well as inside the tumor cell clusters.

**Figure 3:**
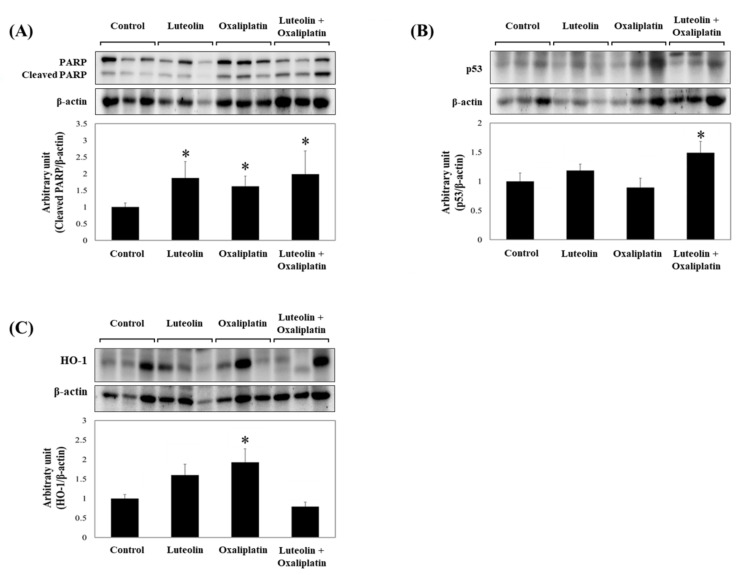
Relative protein levels in HCT116 xenograft tumor homogenates. (**A**–**C**) HCT116 xenograft tumors were collected from mice treated with luteolin and/or oxaliplatin. The total proteins were extracted from the tumor tissues and subjected to western blotting analysis, which was used to quantify the expression of following proteins: cleaved PARP (**A**), p53 (**B**), and HO-1 (**C**). Densitometric data, presented as the mean ± SD (*n* = 6–7 per group) with representative western blotting bands displayed above each graph. Significant differences between groups at *p* < 0.05 is indicated by an asterisk (*).

Consistently, the level of cleaved PARP in xenograft tumors was significantly increased by the treatment with luteolin and oxaliplatin individually, or in combination (Figure 3A). The combined treatment significantly increased p53 protein expression compared to the control or individual treatments, which was suggestive of apoptotic cell death in the tumor (Figure 3B). Unexpectedly, HO-1 protein expression was apparently unaffected by the combined treatment (Figure 3C). These findings suggest that the combination of luteolin and oxaliplatin promoted p53-dependent apoptosis, concurrently prevented cytoprotective HO-1 induction, and thereby retarded growth of the HCT116 xenograft tumors.

### 3.4. Luteolin-Induced AMPK Inhibition Facilitated the Anticancer Effect of Oxaliplatin in HCT116 Xenograft Tumors

**Figure 4:**
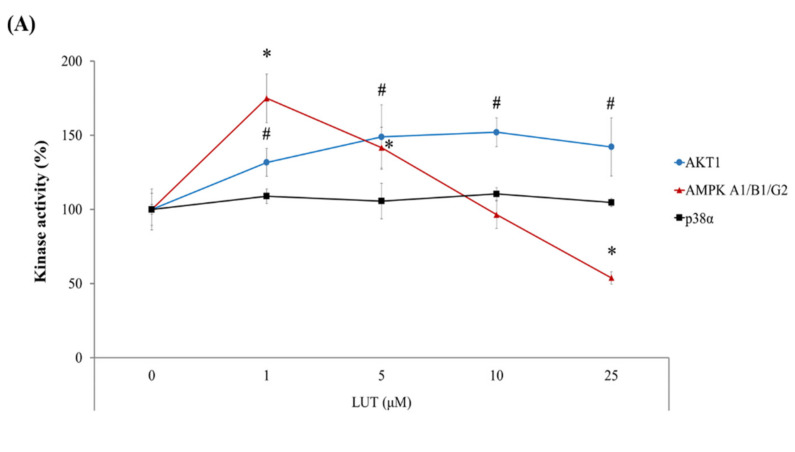

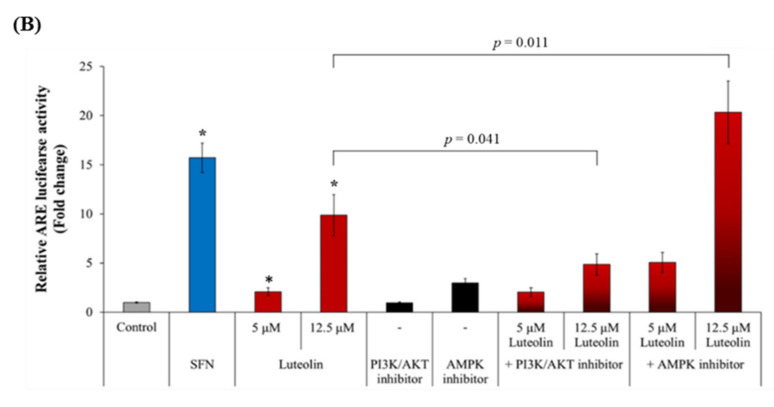
Kinase activities involved in luteolin-induced ARE transcriptional activity. (**A**) Effect of luteolin on the kinase activities. Activities of p38α, AMPK A1/B1/G2, and AKT1 were measured in the presence of luteolin at various concentrations (0.01, 0.1, 1, 5, 10, and 25 μM) and expressed relative to control (without luteolin treatment). (**B**) Relative ARE-luciferase activity in HCT116-ARE cells. Cells were treated with 5 or 12.5 μM luteolin in the absence or presence of LY294002 (a PI3K/AKT inhibitor, 2.5 μM) and compound C (an AMPK inhibitor, 1 μM) for 24 h, and analyzed for ARE-luciferase activity. Sulforaphane (SFN, 3 μM) was used as a positive control to induce ARE activity. The data are expressed as the mean ± SEM (*n* = 6). A significant difference between the groups at *p* < 0.05 was indicated by an asterisk (*). Data are presented as the mean ± SD (*n* = 4). Significant differences compared to the control at *p* < 0.05 are indicated by an asterisk (*) or a hashtag (#).

As shown in the previous section, HO-1 expression was slightly or significantly increased by individual treatment with luteolin or oxaliplatin, but it was barely affected by the combined treatment (Figure 3C). The flavonoid luteolin can enhance HO-1 expression through the upregulation of Nrf2 transcriptional activity, which is reportedly controlled by its upstream protein kinases, including extracellular signal-regulated protein kinase (ERK), c-Jun N-terminal kinase (JNK), p38 MAPK, and PI3K/AKT [23,24,25,26]. To delineate whether luteolin affects the above kinases, the enzyme activity of a set of kinases was screened in the presence of luteolin using a commercially available profiling system. We found that luteolin activated AKT in a concentration-dependent manner and AMPK at relatively low concentrations (5 μM or lower) (Figure 4A). However, luteolin at the concentrations of >10 μM decreased AMPK activity. The activity of p38 MAPK was not influenced by luteolin in the tested concentration range. Thus, it was presumed that relatively high concentrations of luteolin activated the Nrf2/ARE/HO-1 axis, primarily through the activation of AKT and/or the inhibition of AMPK. To test this hypothesis, HCT116-ARE cells were treated with luteolin in the presence of specific inhibitors of AKT and AMPK and the luciferase activity was determined. The treatment of HCT116 cells with luteolin significantly increased ARE-luciferase activity, as expected (Figure 4B). The increased ARE-luciferase activity in the presence of 12.5 μM luteolin was significantly decreased by the inhibition of AKT using LY294002, whereas it was further increased by the inhibition of AMPK using compound C. These results suggest that luteolin promoted the upregulation of Nrf2/ARE/HO-1 axis in HCT116 cells mainly through both AKT activation and AMPK inhibition.

It is noteworthy that luteolin showed contrasting effects on AMPK activity (activation at ≤5 µM luteolin and inhibition at >10 µM luteolin) in HCT116 cells (Figure 4A); however, luteolin-mediated AMPK activation barely affected the transcriptional activity of Nrf2 (Figure 4B).

The association between the PI3K/AKT pathway and the Nrf2/HO-1 axis is well established in various cell types [27,28,29]. Multiple studies have demonstrated that PI3K/AKT activation is essential for the nuclear translocation of Nrf2 [30,31,32] and that many antioxidant phytochemicals, including luteolin, induce Nrf2 activation through the PI3K/AKT signaling pathway [33,34,35]. However, when examining AMPK inhibition by luteolin, an additional western blot showed that HO-1 expression was remarkably increased in HCT116 cells treated with compound C alone or in combination with 12.5 µM luteolin (Supplementary Figure S5). This result supported the negative correlation between AMPK activity and Nrf2/ARE/HO-1 induction. Therefore, our observations suggest that the luteolin-induced activation of Nrf2/ARE/HO-1 axis in HCT116 cells was attributed to the combinatorial effect of AKT activation and AMPK inhibition.

**Figure 5:**
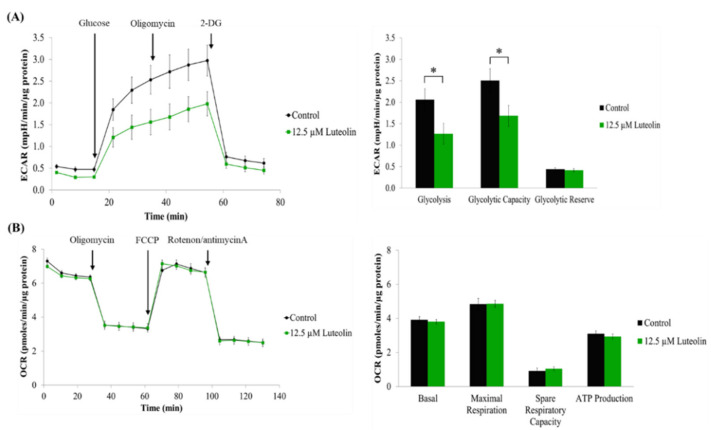
Effect of luteolin on glycolytic capacity and mitochondrial respiration in HCT116 cells. (**A**,**B**) Cells were treated with 12.5 μM of luteolin for 24 h, followed by bioenergetic analysis using the Agilent Seahorse Extracellular Flux Analyzer. ECAR (**A**) and OCR (**B**) values were normalized to the total protein content (ECAR, mpH/min/μg protein; OCR, pmoles/min/μg protein). The data are presented as the mean ± SEM (*n* = 3). Significant differenced compared to the control at *p* < 0.05 are indicated by an asterisk (*).

AMPK is a sensor of cellular energy status [36,37]. The activated AMPK is known to facilitate glucose uptake and glycolysis [38,39]. In cancer cells, AMPK activation stimulates catabolic pathways and suppresses anabolic pathways, thereby increasing tumor growth and contributing to drug resistance [40,41]. When considering the Warburg effect, a hallmark of the metabolic alteration of cancer cells, which rely primarily on glycolysis instead of oxidative phosphorylation for ATP production [42,43,44], the enhanced glycolysis is critically involved in cancer cell survival and propagation [45,46]. To examine if the inhibitory effect of luteolin against AMPK activity alters energy metabolism in HCT116 cells, real-time cell metabolic analysis was performed using an Agilent Seahorse Extracellular Flux Analyzer. We found that luteolin at the AMPK-inhibiting concentration significantly lowered the ECAR, indicating a reduction in glycolysis and glycolytic capacity (Figure 5A). However, the OCR value in luteolin-treated conditions was not statistically different from that for the control, indicating that luteolin exerted only a limited effect on ATP production through mitochondrial respiration in HCT116 cells (Figure 5B). Thus, these findings suggest that AMPK inhibition by luteolin restricted glycolytic metabolism in HCT116 cells, leading to energy depletion and the consequent suppression of cell proliferation.

Although AMPK is widely regarded as a tumor-suppressing factor [47,48], the accumulated data support its pro-tumorigenic role [48,49,50]. It was shown that AMPK activation favors the survival of colorectal cancer cells by promoting NADPH homeostasis under metabolic stress [48,49,50] and that AMPK inhibition enhanced the anticancer effectiveness of oxaliplatin in colorectal cancer models [48]. Currently, the function of AMPK in cancer is considered to be context-dependent [41]. The findings from the present study suggest that luteolin suppressed glycolysis, possibly through the inhibition of AMPK activity by which it contributes to the vulnerability of colorectal cancer cells to oxaliplatin and the subsequent retardation of tumor growth. The precise mechanism of the synergistic anticancer effect of the combined treatment remains to be elucidated.

## 4. Conclusions

Collectively, the results from this study demonstrated that the combined treatment of luteolin and oxaliplatin synergistically inhibited HCT116 xenograft tumor growth in mice by facilitating apoptosis and inhibiting proliferation, most likely through an AMPK-associated mechanism. These findings indicate that a luteolin-rich diet may improve the efficacy of oxaliplatin for colorectal cancer treatment.

## Data Availability

All data generated or analyzed during this study are included in this published article and its Supplementary Materials.

## Supplementary Materials

The following supporting information can be downloaded at: https://www.mdpi.com/article/10.3390/antiox11040626/s1, Figure S1: In vivo experimental scheme; Figure S2: Proportion of apoptotic cells in HCT116 cultures treated with luteolin and oxaliplatin individually or in combination; Figure S3: Body weight changes in HCT116 xenograft mice during the experimental period; Figure S4: Quantification of TUNEL- and PCNA-positive cells in HCT116 xenograft tumor tissue sections; Figure S5: HO-1 protein expression level in HCT116 cells was increased in the presence of AMPK inhibitor.

## References

[1] Rawla P., Sunkara T., Barsouk A. (2019). Epidemiology of Colorectal Cancer: Incidence, Mortality, Survival, and Risk Factors. Prz. Gastroenterol..

[2] Al-Ishaq R.K., Overy A.J., Büsselberg D. (2020). Phytochemicals and Gastrointestinal Cancer: Cellular Mechanisms and Effects to Change Cancer Progression. Biomolecules.

[3] Millen A.E., Subar A.F., Graubard B.I., Peters U., Hayes R.B., Weissfeld J.L., Yokochi L.A., Ziegler R.G., PLCO Cancer Screening Trial Project Team (2007). Fruit and Vegetable Intake and Prevalence of Colorectal Adenoma in a Cancer Screening Trial. Am. J. Clin. Nutr..

[4] Wolin K.Y., Yan Y., Colditz G.A., Lee I.M. (2009). Physical Activity and Colon Cancer Prevention: A Meta-Analysis. Br. J. Cancer.

[5] Larsson S.C., Wolk A. (2006). Meat Consumption and Risk of Colorectal Cancer: A Meta-Analysis of Prospective Studies. Int. J. Cancer.

[6] Shu L., Cheung K.L., Khor T.O., Chen C., Kong A.N. (2010). Phytochemicals: Cancer Chemoprevention and Suppression of Tumor Onset and Metastasis. Cancer Metastasis Rev..

[7] Saw C.L., Cintrón M., Wu T.Y., Guo Y., Huang Y., Jeong W.S., Kong A.N. (2011). Pharmacodynamics of Dietary Phytochemical Indoles I3C and DIM: Induction of Nrf2-Mediated phase II Drug Metabolizing and Antioxidant Genes and Synergism with Isothiocyanates. Biopharm. Drug Dispos..

[8] Tan A.C., Konczak I., Sze D.M., Ramzan I. (2011). Molecular Pathways for Cancer Chemoprevention by Dietary Phytochemicals. Nutr. Cancer.

[9] Yunos N.M., Beale P., Yu J.Q., Huq F. (2011). Synergism from the Combination of Oxaliplatin with Selected Phytochemicals in Human Ovarian Cancer Cell Lines. Anticancer Res..

[10] Guo Y., Su Z.Y., Zhang C., Gaspar J.M., Wang R., Hart R.P., Verzi M.P., Kong A.N. (2017). Mechanisms of Colitis-Accelerated Colon Carcinogenesis and Its Prevention with the Combination of Aspirin and Curcumin: Transcriptomic Analysis Using RNA-Seq. Biochem. Pharmacol..

[11] Santini A., Novellino E. (2018). Nutraceuticals—Shedding Light on the Grey Area Between Pharmaceuticals and Food. Expert Rev. Clin. Pharmacol..

[12] Gutheil W.G., Reed G., Ray A., Anant S., Dhar A. (2012). Crocetin: An Agent Derived from Saffron for Prevention and Therapy for Cancer. Curr. Pharm. Biotechnol..

[13] Menegon S., Columbano A., Giordano S. (2016). The Dual Roles of NRF2 in Cancer. Trends Mol. Med..

[14] Wang X.J., Sun Z., Villeneuve N.F., Zhang S., Zhao F., Li Y., Chen W., Yi X., Zheng W., Wondrak G.T. (2008). Nrf2 Enhances Resistance of Cancer Cells to Chemotherapeutic Drugs, the Dark Side of Nrf2. Carcinogenesis.

[15] Jeong G., Oh J., Kim J.S. (2019). Glyceollins Modulate Tumor Development and Growth in a Mouse Xenograft Model of Human Colon Cancer in a p53-Dependent Manner. J. Med. Food.

[16] Nitti M., Piras S., Marinari U.M., Moretta L., Pronzato M.A., Furfaro A.L. (2017). HO-1 Induction in Cancer Progression: A Matter of Cell Adaptation. Antioxidants.

[17] Pandurangan A.K., Ananda Sadagopan S.K., Dharmalingam P., Ganapasam S. (2014). Luteolin, a Bioflavonoid Inhibits Azoxymethane-Induced Colorectal Cancer Through Activation of Nrf2 Signaling. Toxicol. Mech. Methods.

[18] Li Y., Shen L., Luo H. (2016). Luteolin Ameliorates Dextran Sulfate Sodium-Induced Colitis in Mice Possibly Through Activation of the Nrf2 Signaling Pathway. Int. Immunopharmacol..

[19] Jang C.H., Moon N., Oh J., Kim J.S. (2019). Luteolin Shifts Oxaliplatin-Induced Cell Cycle Arrest at G (0). Nutrients.

[20] Gwon Y., Oh J., Kim J.S. (2020). Sulforaphane Induces Colorectal Cancer Cell Proliferation Through Nrf2 Activation in a p53-Dependent Manner. Appl. Biol. Chem..

[21] Seo H., Oh J., Hahn D., Kwon C.S., Lee J.S., Kim J.S. (2017). Protective Effect of Glyceollins in a Mouse Model of Dextran Sulfate Sodium-Induced Colitis. J. Med. Food.

[22] Feng J., Qin S. (2018). The Synergistic Effects of Apatinib Combined with Cytotoxic Chemotherapeutic Agents on Gastric Cancer Cells and in a Fluorescence Imaging Gastric Cancer Xenograft Model. OncoTargets Ther..

[23] Habtemariam S. (2019). The Nrf2/HO-1 Axis as Targets for Flavanones: Neuroprotection by Pinocembrin, Naringenin, and Eriodictyol. Oxid. Med. Cell. Longev..

[24] Suraweera T.L., Rupasinghe H.P.V., Dellaire G., Xu Z. (2020). Regulation of Nrf2/ARE Pathway by Dietary Flavonoids: A Friend or Foe for Cancer Management?. Antioxidants.

[25] Mo L., Yang C., Gu M., Zheng D., Lin L., Wang X., Lan A., Hu F., Feng J. (2012). PI3K/Akt Signaling Pathway-Induced Heme oxygenase-1 Upregulation Mediates the Adaptive Cytoprotection of Hydrogen Peroxide Preconditioning Against Oxidative Injury in PC12 Cells. Int. J. Mol. Med..

[26] Pae H.O., Kim E.C., Chung H.T. (2008). Integrative Survival Response Evoked by Heme oxygenase-1 and Heme Metabolites. J. Clin. Biochem. Nutr..

[27] Martin D., Rojo A.I., Salinas M., Diaz R., Gallardo G., Alam J., de Galarreta C.M.R., Cuadrado A. (2004). Regulation of Heme Oxygenase-1 Expression Through the Phosphatidylinositol 3-Kinase/Akt Pathway and the Nrf2 Transcription Factor in Response to the Antioxidant Phytochemical Carnosol. J. Biol. Chem..

[28] Jayasooriya R.G.P.T., Park S.R., Choi Y.H., Hyun J.W., Chang W.Y., Kim G.Y. (2015). Camptothecin Suppresses Expression of Matrix Metalloproteinase-9 and Vascular Endothelial Growth Factor in DU145 Cells Through PI3K/Akt-mediated Inhibition of NF-κB Activity and Nrf2-Dependent Induction of HO-1 Expression. Environ. Toxicol. Pharmacol..

[29] Brasil F.B., Bertolini Gobbo R.C., Souza de Almeida F.J., Luckachaki M.D., Dall’Oglio E.L., de Oliveira M.R. (2021). The Signaling Pathway PI3K/Akt/Nrf2/HO-1 Plays a Role in the Mitochondrial Protection Promoted by Astaxanthin in the SH-SY5Y Cells Exposed to Hydrogen Peroxide. Neurochem. Int..

[30] Zipper L.M., Mulcahy R.T. (2003). Erk Activation Is Required for Nrf2 Nuclear Localization During Pyrrolidine Dithiocarbamate Induction of Glutamate Cysteine Ligase Modulatory Gene Expression in HepG2 Cells. Toxicol. Sci..

[31] Nakaso K., Yano H., Fukuhara Y., Takeshima T., Wada-Isoe K., Nakashima K. (2003). PI3K Is a Key Molecule in the Nrf2-Mediated Regulation of Antioxidative Proteins by Hemin in Human Neuroblastoma Cells. FEBS Lett..

[32] Wang L., Chen Y., Sternberg P., Cai J. (2008). Essential Roles of the PI3 Kinase/Akt Pathway in Regulating Nrf2-Dependent Antioxidant Functions in the RPE. Investig. Ophthalmol. Vis. Sci..

[33] Kim J.W., Lim S.C., Lee M.Y., Lee J.W., Oh W.K., Kim S.K., Kang K.W. (2010). Inhibition of Neointimal Formation by Trans-Resveratrol: Role of Phosphatidyl Inositol 3-Kinase-Dependent Nrf2 Activation in Heme oxygenase-1 Induction. Mol. Nutr. Food Res..

[34] Hao Y., Li Y., Liu J., Wang Z., Gao B., Zhang Y., Wang J. (2021). Protective Effect of Chrysanthemum Morifolium cv. Fubaiju Hot-Water Extracts Against ARPE-19 Cell Oxidative Damage by Activating PI3K/Akt-Mediated Nrf2/HO-1 Signaling Pathway. Front. Nutr..

[35] Paredes-Gonzalez X., Fuentes F., Jeffery S., Saw C.L., Shu L., Su Z.Y., Kong A.N. (2015). Induction of NRF2-Mediated Gene Expression by Dietary Phytochemical Flavones Apigenin and Luteolin. Biopharm. Drug Dispos..

[36] Lin S.C., Hardie D.G. (2018). AMPK: Sensing Glucose as Well as Cellular Energy Status. Cell Metab..

[37] Hardie D.G., Ross F.A., Hawley S.A. (2012). AMPK: A Nutrient and Energy Sensor That Maintains Energy Homeostasis. Nat. Rev. Mol. Cell Biol..

[38] O’Neill H.M. (2013). AMPK and Exercise: Glucose Uptake and Insulin Sensitivity. Diabetes Metab. J..

[39] Long Y.C., Zierath J.R. (2006). AMP-Activated Protein Kinase Signaling in Metabolic Regulation. J. Clin. Investig..

[40] Wang Z., Liu P., Chen Q., Deng S., Liu X., Situ H., Zhong S., Hann S., Lin Y. (2016). Targeting AMPK Signaling Pathway to Overcome Drug Resistance for Cancer Therapy. Curr. Drug Targets.

[41] Zadra G., Batista J.L., Loda M. (2015). Dissecting the Dual Role of AMPK in Cancer: From Experimental to Human Studies. Mol. Cancer Res..

[42] Liberti M.V., Locasale J.W. (2016). The Warburg Effect: How Does It Benefit Cancer Cells?. Trends Biochem. Sci..

[43] Warburg O. (1925). The Metabolism of Carcinoma Cells. J. Cancer Res..

[44] Ganapathy-Kanniappan S., Geschwind J.F. (2013). Tumor Glycolysis as a Target for Cancer Therapy: Progress and Prospects. Mol. Cancer.

[45] Pfeiffer T., Schuster S., Bonhoeffer S. (2001). Cooperation and Competition in the Evolution of ATP-Producing Pathways. Science.

[46] Shiratori R., Furuichi K., Yamaguchi M., Miyazaki N., Aoki H., Chibana H., Ito K., Aoki S. (2019). Glycolytic Suppression Dramatically Changes the Intracellular Metabolic Profile of Multiple Cancer Cell Lines in a Mitochondrial Metabolism-Dependent Manner. Sci. Rep..

[47] Liu C., Liu Q., Yan A., Chang H., Ding Y., Tao J., Qiao C. (2020). Metformin Revert Insulin-Induced Oxaliplatin Resistance by Activating Mitochondrial Apoptosis Pathway in Human Colon Cancer HCT116 Cells. Cancer Med..

[48] Wang Y.N., Lu Y.X., Liu J., Jin Y., Bi H.C., Zhao Q., Liu Z.X., Li Y.Q., Hu J.J., Sheng H. (2020). AMPKα1 Confers Survival Advantage of Colorectal Cancer Cells Under Metabolic Stress by Promoting Redox Balance Through the regulationRegulation of Glutathione Reductase Phosphorylation. Oncogene.

[49] Jeon S.M., Hay N. (2015). The Double-Edged Sword of AMPK Signaling in Cancer and Its Therapeutic Implications. Arch. Pharm. Res..

[50] Jeon S.M., Chandel N.S., Hay N. (2012). AMPK Regulates NADPH Homeostasis to Promote Tumour Cell Survival During Energy Stress. Nature.

