# Supplementary material for: Luteolin Synergistically Enhances Antitumor Activity of Oxaliplatin in Colorectal Carcinoma via AMPK Inhibition
Source: Antioxidants (Basel). 2022 Mar 24;11(4):626. doi: 10.3390/antiox11040626 (PMC9030203; doi:10.3390/antiox11040626)
Supplement: Supplementary file 1 [file antioxidants-11-00626-s001.zip › antioxidants-1625734-supplementary.pdf]

antioxidants

MDPI

Supplementary Materials: Luteolin Synergistically Enhances Antitumor Activity of Oxaliplatin in Colorectal Carcinoma via AMPK Inhibition

Chan Ho Jang [1,†], Nayoung Moon [1,†], Jinkyung Lee [1], Min Jeong Kwon [1], Jisun Oh [2,*] and Jong-Sang Kim [1,*]

[1] School of Food Science and Biotechnology, Kyungpook National University, Daegu 41566, Korea; cksghwkd7@gmail.com (C.H.J.); moonna1008@naver.com (N.M.); wlsrud9526@naver.com (J.L.); rnjsalswjd1005@naver.com (M.J.K.)
[2] New Drug Development Center, Daegu-Gyeongbuk Medical Innovation Foundation, Daegu 41061, Korea
* Correspondence: joh@kmedihub.re.kr (J.O.; Tel. +82-53-790-5236); vision@knu.ac.kr (J.-S.K.; Tel.: +82-53-950-5752)
† These authors contributed equally.

Suppl. Figure S1. *In vivo* experimental scheme.

Suppl. Figure S2. Proportion of apoptotic cells in HCT116 cultures treated with luteolin and oxaliplatin individually or in combination.

Suppl. Figure S3. Body weight changes of HCT116 xenograft mice during the experimental period.

Suppl. Figure S4. Quantification of TUNEL- and PCNA-positive cells in HCT116 xenograft tumor tissue sections.

Suppl. Figure S5. HO-1 protein expression level in HCT116 cells was increased in the presence of AMPK inhibitor.

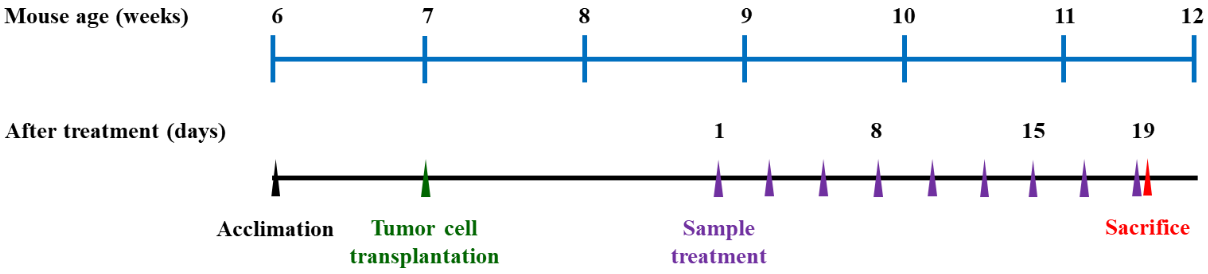

Supplementary Figure S1. *In vivo* experimental scheme.

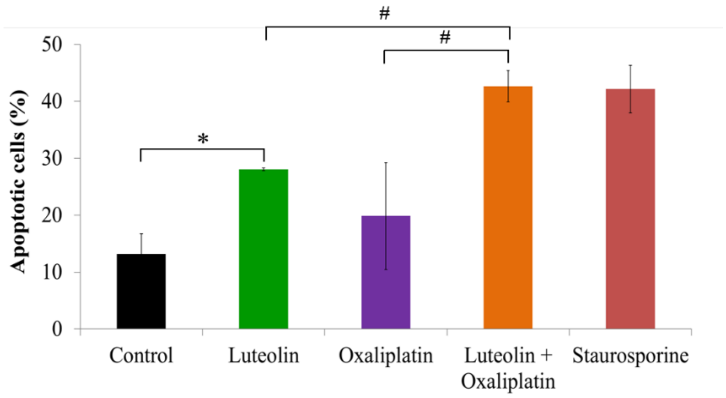

Supplementary Figure S2. Proportion of apoptotic cells in HCT116 cultures treated with luteolin and oxaliplatin individually or in combination. Cells were treated with 25 µM of luteolin with or without 1 µM of oxaliplatin for 24 h. Staurosporine (2 µM, 4 h) was used as a positive control to induce apoptosis. Apoptotic cells were detected using a flow cytometer. Data are presented as mean ± SD (n = 2). A significant difference between the groups at $p < 0.05$ was indicated by an asterisk (*) or a hashtag (#).

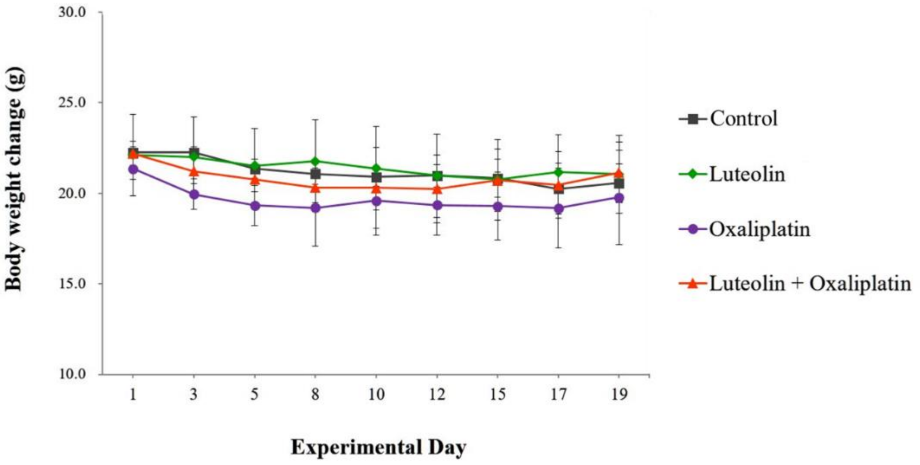

Supplementary Figure S3. Body weight changes of HCT116 xenograft mice during the experimental period. HCT116 cells were subcutaneously transplanted into the left and right flanks of BALB/c nude mice. Body weight of mice was regularly monitored and measured until sacrifice. Data are expressed as mean ± SD (n = 4 per group).

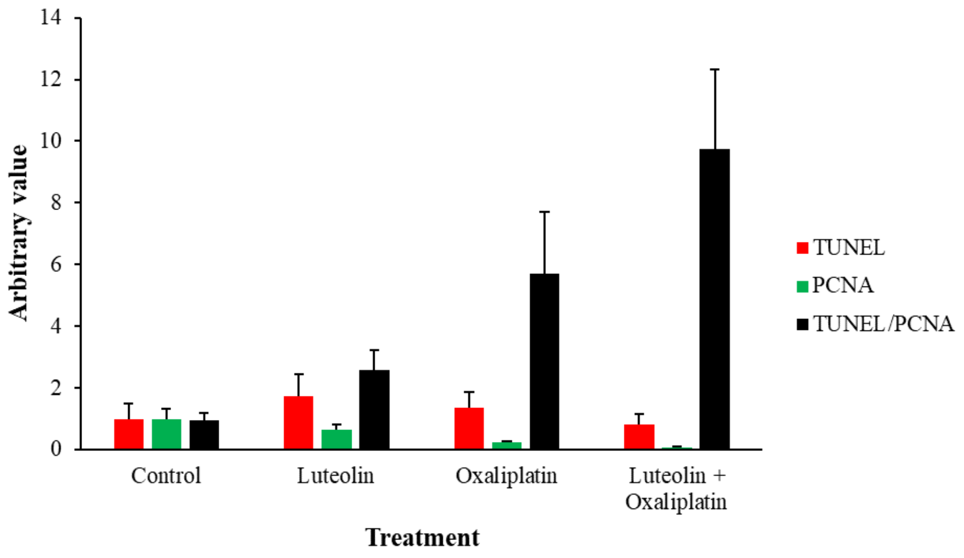

Supplementary Figure S4. Quantification of TUNEL- and PCNA-positive cells in HCT116 xenograft tumor tissue sections. Luteolin treatment slightly increased the portion of TUNEL-positive cells, and oxaliplatin treatment considerably decreased the portion of PCNA-positive cells. The ratio of TUNEL-stained population over PCNA-stained population was remarkably increased by the combined treatment of luteolin and oxaliplatin.

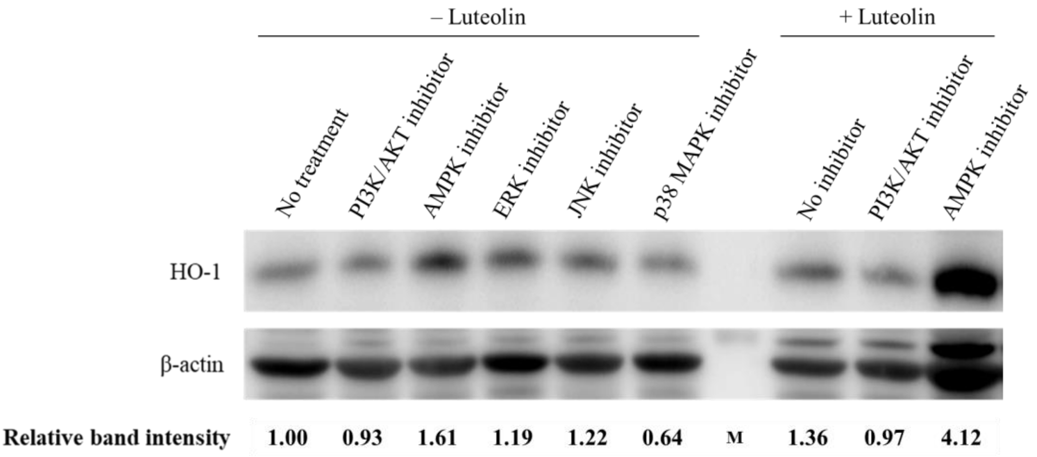

Supplementary Figure S5. HO-1 protein expression level in HCT116 cells was increased in the presence of AMPK inhibitor. HCT116 cells were treated with luteolin at 12.5 μM without or with PI3K/AKT inhibitor (LY294002, 2.5 μM), AMPK inhibitor (compound C, 1 μM), ERK inhibitor (PD98059, 5 μM), JNK inhibitor (SP600125, 1 μM), or p38 MAPK inhibitor (SB203580, 20 μM) for 24 h and then subjected to Western blotting analysis. HO-1 expression in HCT116 cells was remarkably increased when treated with compound C alone or in combination with luteolin, compared to the control. M, protein size marker.
